# *Bacillus anthracis* Peptidoglycan Stimulates an Inflammatory Response in Monocytes through the p38 Mitogen-Activated Protein Kinase Pathway

**DOI:** 10.1371/journal.pone.0003706

**Published:** 2008-11-12

**Authors:** Marybeth Langer, Alexander Malykhin, Kenichiro Maeda, Kaushik Chakrabarty, Kelly S. Williamson, Christa L. Feasley, Christopher M. West, Jordan P. Metcalf, K. Mark Coggeshall

**Affiliations:** 1 Immunobiology and Cancer Program, Oklahoma Medical Research Foundation, Oklahoma City, Oklahoma, United States of America; 2 Pulmonary and Critical Care Division, Department of Medicine, University of Oklahoma Health Sciences Center, Oklahoma City, Oklahoma, United States of America; 3 Free Radical Biology & Aging Program, Oklahoma Medical Research Foundation, Oklahoma City, Oklahoma, United States of America; 4 Oklahoma Center for Medical Glycobiology, Department of Biochemistry and Molecular Biology, University of Oklahoma Health Sciences Center, Oklahoma City, Oklahoma, United States of America; University of Cambridge, United Kingdom

## Abstract

We hypothesized that the peptidoglycan component of *B. anthracis* may play a critical role in morbidity and mortality associated with inhalation anthrax. To explore this issue, we purified the peptidoglycan component of the bacterial cell wall and studied the response of human peripheral blood cells. The purified *B. anthracis* peptidoglycan was free of non-covalently bound protein but contained a complex set of amino acids probably arising from the stem peptide. The peptidoglycan contained a polysaccharide that was removed by mild acid treatment, and the biological activity remained with the peptidoglycan and not the polysaccharide. The biological activity of the peptidoglycan was sensitive to lysozyme but not other hydrolytic enzymes, showing that the activity resides in the peptidoglycan component and not bacterial DNA, RNA or protein. *B. anthracis* peptidoglycan stimulated monocytes to produce primarily TNFα; neutrophils and lymphocytes did not respond. Peptidoglycan stimulated monocyte p38 mitogen-activated protein kinase and p38 activity was required for TNFα production by the cells. We conclude that peptidoglycan in *B. anthracis* is biologically active, that it stimulates a proinflammatory response in monocytes, and uses the p38 kinase signal transduction pathway to do so. Given the high bacterial burden in pulmonary anthrax, these findings suggest that the inflammatory events associated with peptidoglycan may play an important role in anthrax pathogenesis.

## Introduction

During early stages of infections *B. anthracis*, spores are engulfed by phagocytic cells which serve to traffic the organism to the lymphatic system and bloodstream where severe disease ensues due to rapid growth of the organism. Macrophages are thought to be critical to both the early and late stages of this disease, with distinct contributions to infection and progression of disease. Indeed, when mice are depleted of macrophages and challenged with *B. anthracis* spores, the animals have a shorter mean time to death than those with a normal compliment of macrophages [1]. These studies suggest macrophages play a major role in defending the host against the infection.

On the other hand, *B. anthracis* vegetative bacteria profoundly expand during active infection, achieving 10^8^–10^9^ organisms per milliliter of blood; [2], [3]. The expansion is such that every organ shows the presence of vegetative bacteria [4], as well as pleural effusions [4] and cerebrospinal fluid [5]. The median incubation from time of exposure to fulminate disease is rapid [4–6 days; [5]] and infected patients have a poor prognosis, such that ∼40% of cases are fatal even with aggressive antibiotic therapy [4], [5], [6]. Therefore, given the high bacterial burden, it is also reasonable to hypothesize that macrophages may contribute to sepsis and death by releasing proinflammatory cytokines. Although previous studies have focused extensively on the macrophage's role in early interactions with *B. anthracis*, very little is known about the influence of *B. anthracis* on macrophage function during later stages of disease. In particular the contribution of subcellular components of *B. anthracis* to septic shock have not been elucidated.

Human septic shock is associated with increased levels of the proinflammatory cytokines interleukin (IL)-1, tumor necrosis factor α (TNFα) and IL-6 [7]. These cytokines are released when human monocytes are stimulated with various Gram-negative and Gram-positive bacteria strains [8]. Monocytes may respond to the vegetative bacteria during *B. anthracis* infections by releasing proinflammatory cytokines that contribute to septic shock and death. Yet, the *B. anthracis* factors critical to proinflammatory cytokine production have not been fully identified.

*B. anthracis* carries two virulence factors needed for toxemia and/or for full pathogenicity. First, a poly-D-glutamic acid-containing capsule provides *B. anthracis* with resistance to phagocytosis by host myeloid cells [9]. The capsule is unique to *B. anthracis* and is carried on a plasmid pXO2 [10]. Second, a tripartite exotoxin is encoded on the plasmid pXO1. The proteins that comprise the toxin are protective antigen (PA), lethal factor and edema factor. PA binds host cells and to lethal and edema factors to form lethal toxin (LT) and edema toxin (ET) which are internalized into host cells [11]. LT is a zinc metalloprotease that cleaves mitogen-activated protein kinase kinases and thus interferes with signal transduction processes leading to new gene expression [12]. ET acts an adenylate cyclase that causes an elevation in intracellular cyclic AMP, a condition that has been shown to reduce the phagocytic ability of neutrophils [13].

Although injection of animals with the purified toxins of *B. anthracis* cause death [14], the physiological relevance of the toxin administered as a purified protein to death that accompanies human infection by *B. anthracis* is not clear. First, cells within the infected animals contact more than just the toxin proteins of *B. anthracis*. Gram-positive organisms contain a rigid peptidoglycan (PGN) cell wall that is reported to engage Toll-like receptors (TLR) 2 and 6 [15], the transmembrane peptidoglycan-recognition proteins [16], and the nucleotide-binding oligomerization domain (NOD) 1 and 2 proteins [17], [18], [19]. Agents that bind these receptors on innate immune cells stimulate the production of proinflammatory cytokines including IL-8 and TNFα [15], [20]. Second, *B. anthracis* strains lacking LT and ET, when used to infect mice, show a LD_50_ and a mean time to death that is not significantly different from that of *B. anthracis* strains having functional LT and ET [11]. Because of the high bacterial load in infected individuals and the ability of other Gram-positive PGN species to stimulate inflammation, the PGN of *B. anthracis* is a potential virulence factor that has not been investigated for its role in inflammation leading to septic shock. These findings in animal models raise the possibility that septic shock may also be relevant to human pathology. Therefore, the endogenous toxins present in the *B. anthracis* cell wall may be important virulence factors.

The chemical composition of and host response to *B. anthracis* PGN has not been characterized. PGN is a large glycan polymer composed of alternating N-acetylglucosamine (GlcNac) and N-acetylmuramic acid (MurNac) residues joined by short (4–5 L- and D-amino acids, [21]) stem peptides [21]. The stem peptides can be linked to each other either directly or through more complex polypeptide bridges [21], [22]. The glycan strand, the particular stem peptide residues and their length, and the presence and location of the interpeptide bridges can vary between species [22]. For example, *S. aureus* PGN shows a pentaglycine polypeptide linking the stem peptides, the presence of which renders the PGN sensitive to the protease lysostaphin [23].

A polysaccharide may be covalently bound to some Gram-positive PGN molecule. The polysaccharide can be released by hydrogen fluoride (HF)-mediated hydrolysis. The presence of the polysaccharide can support the proinflammatory activity of PGN of some Gram-positive species [24]. The PGN of *B. anthracis* is known to contain a polysaccharide but its role in inflammation has not been studied [25]. Additionally, lipoteichoic acid (LTA) and teichoic acids may be associated with the PGN of Gram-positive organisms. LTA is a negatively charged glycolipid with a polyglycerol phosphate backbone and is anchored to the cell membrane by the lipid unit [26]. LTA is a potent inducer of proinflammatory cytokine release by innate immune cells [27].

Given the inflammation-inducing properties of PGN and its associated components, we hypothesized that peptidoglycan of *B. anthracis* has a role in the sepsis and death of the host that occurs in the infection. This hypothesis predicts that PGN from *B. anthracis* promotes the induction of proinflammatory cytokines in human peripheral blood (PB) cells associated with the inflammation during infections. To test this prediction, we purified the uncharacterized *B. anthracis* PGN from vegetative bacteria. Using this preparation, we found that *B. anthracis* PGN stimulated TNFα production from whole blood, and that the monocytes were the sole responding population. The biological activity in *B. anthracis* PGN was not sensitive to DNase, RNase, or various proteases nor to reagents that neutralize Gram-negative endotoxin. We also found that PGN-mediated activation of p38 MAP kinase was essential in the response. Our data indicate that PGN from *B. anthracis* can initiate TNFα production through a pathway involving p38 MAP kinase. These findings are consistent with the notion that *B. anthracis* pathophysiology can be at least in part due to septic shock of the host. These findings have important implications for both vaccine development and treatment of human disease.

## Materials and Methods

### Materials

*B. anthracis* crude cell wall extract was purchased from List Biological Laboratories (Campbell, CA). Trypsin Gold, mass spectrometry grade was purchased from Promega. DNase 1, Ultra Pure *E. coli* lipopolysaccharide (LPS) and Polymixin B was purchased from Invivogen (San Diego, CA). RNase A was purchased from Qiagen. Brefeldin A, and antibodies to human CD14, CD16b, CD3, CD19 and TNFα were purchased from eBioscience, San Diego, CA. The Cambrex Limulus amebocyte lysate assay was purchased from Lonza. The phospho-protein antibodies used for intracellular staining were purchased from Cell Signaling (Danvers, MA) as Alexafluor 488 or Alexafluor 647 conjugates, and were phospho-p38 MAP kinase (Thr180/Try182), phospho-p44/42 (T202/Y204) and phospho-SAPK/JnK (Thr183/Tyr185). MAP kinase inhibitors SB202190, SP600125 and PD98095 were purchased from Calbiochem (San Diego, CA). *Bacillus subtilis* lipoteichoic acid was purchased from Sigma-Aldrich (Milwaukee, WI).

### Isolation and analysis of *B. anthracis* PGN

*B. anthracis* Delta Sterne strain (lacking capsule and toxins) PGN was prepared by the method described by Popov *et al.* [28], with modifications as described [29]. Endotoxin-free water used throughout. Tryptic soy broth plates were spread with 0.1 ml of an overnight culture of *B. anthracis*. The plates were incubated again overnight at 37°C and the bacteria were scraped from plates with a small amount (∼50 mls) of cold tryptic soy broth media. Bacteria were washed in endotoxin-free water, resuspended in 5 ml 8% SDS and boiled for 30 minutes. The lysed cells were centrifuged and the pellet was washed with endotoxin-free water to removes SDS. The resulting material was resuspended in DNase1 buffer with 40 Units of DNase 1 and 7 Units RNase A and incubated 15 min at room temperature. The DNase and RNase were extracted by a second boiling in 4% SDS followed by three endotoxin-free water washes, one wash with 2 M NaCl and six additional endotoxin-free water washes. The final pellet was suspended in endotoxin-free water and boiled for 5 minutes. The pellet was dried, weighed, resuspended in endotoxin-free water and stored at 4°C. *Bacillus subtilis* (ATCC 6051) PGN was extracted by the same method, except that heat killed *B. subtilis* (65°C, 1 hour) was used as the starting material.

For monosaccharide analysis, PGN was hydrolyzed with either 6 M HCl to quantitate amino sugars [30], or 4 M trifluoroacetic acid (TFA) for 4 hrs at 80° C. [31] to quantitate neutral sugars. Samples were dried by vacuum centrifugation and chromatographed isocratically on a CarboPac PA-1 Dionex column in 15 mM NaOH. Sugars were quantitated by pulsed amperometric detection using response factors determined from weighed amounts of known sugars.

### Culture of peripheral blood

Heparinized human PB was drawn by venipuncture with the signed informed consent of human subjects according to a protocol approved by the Oklahoma Medical Research Foundation Institutional Review Board. Blood was diluted 1∶3 with Dulbecco's Modified Eagle's Medium. Diluted whole blood was cultured for all experiments in non-tissue culture 24 well plates or micro-centrifuge tubes.

### Measurement of cytokines in PB supernatant

The PGN preparation, at concentrations described in the text, was added to diluted blood in 24 well culture plates. The plate was incubated at 37°C for various time periods and centrifuged for collection of supernatant. Sandwich enzyme-linked immunosorbant assays (ELISA) for TNFα were performed on the supernatants as described [32]. Multiplex bead immunoassays were performed on the supernatants using a Biosource Human Cytokine 25-Plex (Camarillo, CA). The assay was analyzed on a Luminex 100 with version 2.2 software (Luminex, Austin, TX). Standard curves for cytokines were fit using Applied Cytometry Systems STarStation 2.0 (Plano, TX).

### Flow cytometry analysis

The surface markers CD14 and CD16b were tested for their ability to identify monocytes by sorting cells based on CD14/CD16b, CD3 and CD19 intensity, preparing slides, staining with May-Grunwald Giemsa to confirm identity by morphology. The cells were resuspended in 200 µl fluorescence-activated cell sorter buffer [phosphate-buffered saline (PBS), containing 3% fetal calf serum, 0.1% NaN_3_] and stained with antibodies to surface markers (CD14, CD16b, CD19, and CD3). Cells were gated or sorted based on forward scatter (FSC) and side scatter (SSC) and positive fluorescent surface markers. A mutually exclusive lymphocyte gate and monocyte/neutrophil were drawn on the FSC vs. SSC plot. These serial gates (FSC/SSC and surface marker-positive fluorescent gates) were used in all subsequent experiments to identify the various blood cell subpopulations. For analysis of cytokines, 3 µg/ml Brefeldin A was added to cultures; in some experiments Polymyxin B was added at a concentration of 10 µg/ml. After the indicated stimulation time, the blood cells were transferred to microfuge tubes and the plates were washed with PBS/Brefeldin A, 0.02% EDTA to collect adherent cells. Purified human IgG (0.1 mg/ml) was added to block IgG receptors present on myeloid and lymphoid cells. Cells were washed with PBS/Brefeldin A and fixed for 20 minutes at room temperature with 100 µl PBS containing 2% formaldehyde. The samples were permeabilized with 0.5% saponin before staining with anti-TNFα antibodies (2 µg/ml). Data were collected on 10,000 cells per sample. The percent of TNFα positive cells was calculated using number positive cells/total number of cells. For intracellular phospho-protein staining, PB was stimulated with PGN or LPS (1 µg/ml) and carried out essentially as described [33]. Briefly, cells were fixed with 4% formaldehyde, surface stained with CD14-PE in buffer containing 0.2 mg/ml purified human IgG to block IgG receptors, resuspended in 1 ml permeabilization buffer (50% methanol; 0.5× PBS), and stained with anti-phospho MAP kinase antibodies in buffer containing 0.2 mg/ml human IgG.

### Treatment with modifying enzymes

The purified *B. anthracis* PGN was incubated for 24 hours at 37°C with each of the following modifying enzymes or buffers: lysozyme (Sigma) 66 µg/ml; 2,758,000 U/ml); lysostaphin (Sigma) 150 µg/ml; ≥45 U/ml; DNase 1; 100 U/ml; RNase A 250 µg/ml, trypsin 2.6 µg/ml; proteinase K (Sigma), 50 µg/ml. The buffer for the lysozyme, lysostaphin, and trypsin digests was 0.02 M Tris-HCL, 0.05 M KCL (pH 8.4). For proteinase K buffer, 1 mM Ca^2+^ was added in the in the form of CaCl_2_. After incubation, the hydrolytic enzymes were heat-inactivated 98°C 10 min, stored at 4°C, and the samples were used directly in the assays without further manipulation.

### Isolation of polysaccharide from PGN

HF hydrolysis of phosphodiester bonds and ethanolic precipitation of the released polysaccharide was performed as described [34] with modifications. To 1.3 mg dried PGN, 176 µl of 48% HF was added and the sample was sonicated 8 minutes to suspend the PGN. The sample was incubated at 4°C for 24 hours, centrifuged, and the HF-soluble supernatant containing the polysaccharide was removed to a new tube. The supernatant was mixed with 100% ethanol to precipitate the polysaccharide and centrifuged. The polysaccharide pellet was washed three times with 100% ethanol and dissolved in endotoxin-free water. The HF-insoluble PGN was washed 3 times with endotoxin-free water. Both the HF-soluble and -insoluble samples were air dried and redissolved in endotoxin-free water to their original volume. The optical density at 600 nm was measured. PGN concentration was determined using a standard curve of commercial *B. anthracis* cell wall (List Labs) *versus* OD_600_.

### Extraction of LTA

The procedure to purify LTA from *B. anthracis* was performed as described [27] with the following modifications. *B. anthracis* Sterne strain was grown to an OD of 0.6 in tryptic soy broth, resuspended in 0.1 M sodium citrate buffer pH 4.7 and sonicated for 10 minutes before adding an equal volume of butanol and mixing at room temperature for 30 minutes. After centrifugation, the aqueous phase was lyophilized. Further purification of extracted LTA was by hydrophobic interaction chromatography as described [35], using a HiTrap™ Octyl Sepharose FF 1 ml column (Pharmacia). A linear gradient method was used with solvent A (15% isopropanol, 100 mM ammonium acetate pH 4.7) and solvent B (60% isopropanol, 100 mM ammonium acetate, pH 4.7). One ml fractions were collected. The phosphate content of PGN preparations and fractions from LTA purification was measured as described [36].

### Extraction of RNA and Quantitative PCR

Diluted PB, prepared as above, was stimulated for 2 hours with PGN at 10 µg/ml. Samples were extracted with Trizol Reagent (Invitrogen, Carlsbad, CA) and cDNA was synthesized using Superscript First Strand Synthesis System for PCR (Invitrogen) using 2 µg total RNA. Real-time quantitative PCR was performed on 20 µl volumes using RT-PCR primer set for human TNF or RT-PCR primer set for human ACTB (SuperArray Bioscience Corporation, Frederick, MD), SYBR Green PCR Master Mix (Applied Biosystems, Foster City, CA) and first strand DNA template. DNA was amplified on the Real Time PCR System 7500 (Applied Biosystems, Foster City, CA): 10 mins at 95°C, 40 cycles of 95°C for 15 secs, 60°C for 1 min. Analysis of relative gene expression was performed as described earlier [37].

### Statistical analysis

Where applicable, the data were expressed as the means±standard errors of the means (SEM). Statistical significance was determined by one-way analysis of variance (ANOVA) with Bonferroni post test or by paired or unpaired t test. A p value of <0.05 was considered significant.

## Results

### Analysis of *B. anthracis* PGN

**Figure 1:**
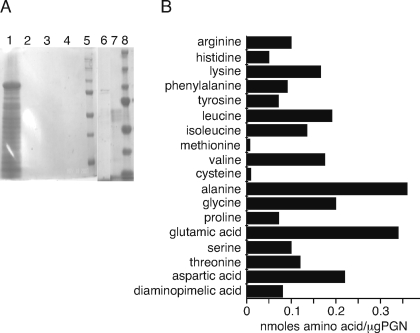
Purified peptidoglycan from *B. anthracis* lacks noncovalently bound proteins. (A) Silver-stained SDS PAGE showing with samples from purification steps and comparison to commercial preparation. Lane 1: bacteria after harvest, lane 2: equivalent sample of extracted PGN, lane 3: molecular weight marker. (B) Amino acid content of *B. anthracis* PGN.

We isolated PGN from *B. anthracis* cell walls by sequential SDS extractions, hydrolysis of contaminating nucleic acids with RNase and DNase, and high salt extractions. We first performed SDS/PAGE analysis of the material under reducing conditions (5% β-mercaptoethanol) to look for non-covalently bound proteins that might contaminate the PGN preparation. The silver-stained gel is shown in Figure 1A and demonstrates the complete absence of proteins (lane 2). However, bacterial proteins were readily detected in lysates of whole *B. anthracis* (Figure 1A lane 1). The data suggest that the PGN preparation does not contain associated bacterial protein, nor protein linked by disulfide bonds.

The purified PGN was assessed for amino acid content by acid hydrolysis and HPLC analysis of the resulting amino acids (Figure 1B). The most abundant amino acids (defined as >0.2 nmoles/µgram of PGN) were alanine (0.36), glutamic acid (includes glutamine) (0.34), aspartic acid (includes asparagine) (0.22), and glycine (0.20) which are typical of the stem peptide and polypeptide bridges in other Gram-positive organisms. We did not distinguish L- and D-amino acids although both are reported to be present in stem peptides and bridges [21]. The source of the minor amino acids is not clear and could reflect the complexity of the *B. anthracis* stem peptide, the presence of an interpeptide bridge, or contaminating covalently-bound membrane protein. Alanine and glutamic acid are abundant because they are major components of stem peptides in other Gram-positive PGN species [21]. Glycine may be abundant because it can be substituted in the α-carboxyl unit of a D-glutamic acid residue of a stem peptide, as had been shown for *Micrococcus leuteus* [21]. The presence of a significant amount of aspartic acid suggests either a substitution in a stem peptide or a polypeptide bridge containing aspartic acid similar to that found in many species [21]. The other minor amino acids may represent substitutions in stem peptides or may be bound to stem peptide residues. In experiments described below we have subjected this PGN preparation to various enzymatic treatments, including proteinase K. We found that the amino acids present in the PGN are essentially identical, before and after PGN digestion with proteinase K (not shown).

PGN from *B. anthracis* and other Gram-positive organisms contain a covalently-bound polysaccharide that is sensitive to HF digestion [25]. To analyze the monosaccharides of the PGN preparation, we treated PGN with HF to cleave phosphodiester linkages. The digested material was centrifuged to separate the HF-insoluble PGN fraction from the HF-soluble polysaccharide fraction. These fractions were hydrolyzed in HCl to quantitate amino sugars and TFA to quantitate neutral sugars. The monosaccharides were resolved using high pH anion exchange chromatography and electrochemical detection. We analyzed the *B. anthracis* PGN preparation for monosaccharide composition before and after HF-mediated removal of the polysaccharide, followed by separation of the HF-insoluble PGN fraction from the HF-soluble polysaccharide fraction (Table 1). As expected, muramic acid was detected only in the insoluble fraction, indicating that all of the PGN remained insoluble after HF treatment. By comparison, the majority of galactose and mannosamine (60%) was recovered in the soluble fraction. Because galactose is exclusive to the polysaccharide in Gram-positive organisms [25], this indicates that the majority of polysaccharide was released by HF treatment. In addition to the monosaccharides listed in Table 1, galactosamine was detected at 0.044 nmoles/µg PGN. Because galactosamine concentration was low relative to the other monosaccharides, we could not conclusively determine whether galactosamine is a constituent of the polysaccharide or of the PGN backbone. Other monosaccharides below this threshold were not detected.

**Table 1:** Monosaccharide composition of HF-hydrolyzed PGN expressed as nmoles/µg of PGN (dry weight).

	HF-polysaccharide fraction	HF-PGN solid fraction	Total HF hydrolyzed fractions^3^
Galactose^1^	0.59	0.35	0.94
Glucose^1^	0.52	0.24	0.76
Mannosamine^2^	0.21	0.15	0.36
Glucosamine^2^	0.98	1.9	2.9
Muramic acid^2^	0.03	1.4	1.4

1 Neutral sugar analysis based on Trifluoroacetic acid hydrolysis.

2 Amino sugar analysis based on HCl hydrolysis.

3 Total derived by mathematical addition of HF-hydrolyzed fractions.

### TNFα is the predominant cytokine that increases in peripheral blood cells in response to increasing stimulation with PGN

**Figure 2:**
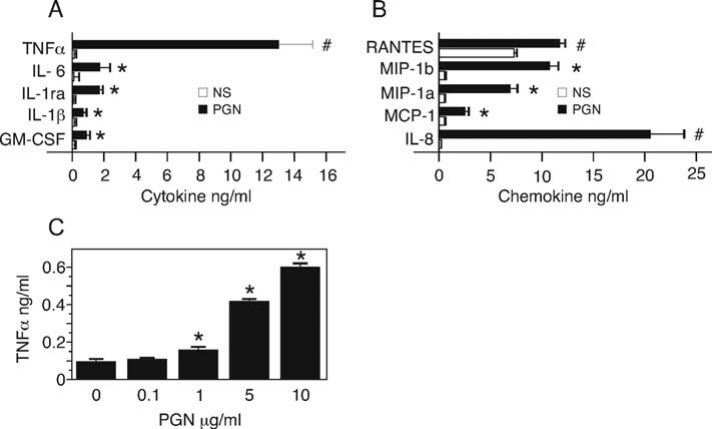
TNFα is the major cytokine in PB produced in response to *B. anthracis* PGN. PB was stimulated with either 10 µg/ml or increasing doses of PGN for 6 hours. (A, B). Secreted cytokines (A) and chemokines (B) in PB supernatants were measured by human multiplex bead immunoassay. Data are expressed as mean±SEM of 3 replicate wells. Statistical significance was determined for stimulated PB (PGN; 10 µg/ml) *versus* unstimulated (NS) by unpaired, one-tailed t test; *, p<0.001 *versus* 0 PGN; #, p<0.05 *versus* 0 PGN (C) Dose-response of secreted TNFα in supernatant measured by ELISA. The data are expressed as mean±SEM of 3 replicate wells. Statistical significance was determined by ANOVA with Bonferroni post test. *, p<0.001 *versus* 0 PGN.

To investigate the inflammatory response of human peripheral blood (PB) cells to the *B. anthracis* vegetative PGN component of the *B. anthracis* cell wall, we stimulated diluted whole human blood for 6 hours with *B. anthracis* PGN and with LPS as a control. We measured cytokines in the PB supernatant induced by stimulating with PGN at 10 µg/ml. We found (Figure 2) that PGN induced production of the cytokines TNFα, IL-6, IL-1 receptor agonist (IL-1ra), IL-1β and granulocyte-macrophage colony stimulation factor (GM-CSF) and the chemokines IL-8, monocyte inflammatory protein (MIP) 1α and 1β, macrophage chemotactic protein (MCP)-1, and Regulation upon Activation Normal T cell Expressed and Secreted (RANTES). Since TNFα was the major proinflammatory cytokine produced by PGN, we measured the production of TNFα to increasing doses of PGN (Figure 2C). For doses ≥1.0 µg/ml, the TNFα concentration in the supernatant was significantly different than in the unstimulated samples (P<0.001; marked by *). Thus, cells within whole PB respond to *B. anthracis* PGN by producing proinflammatory cytokines, predominantly TNFα, and a number of chemokines that are known to recruit innate immune cells. Because our hypothesis predicts the induction of proinflammatory cytokines and TNFα is an important proinflammatory mediator in disease [38], we focused on the source of TNFα and the functional relationship between *B. anthracis* PGN and the induction of TNFα.

### Monocytes, not neutrophils or lymphocytes, produce TNFα in response to PGN

**Figure 3:**
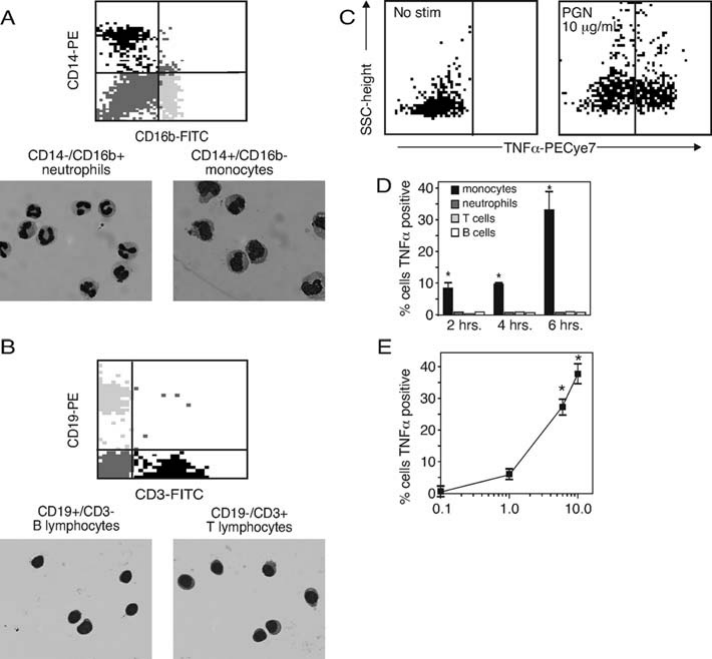
Monocytes produce TNFα in response to PGN. (A) Blood cells were sorted by SSC/FSC properties and staining intensity of fluorescent surface markers CD14, CD16b, CD3, CD19. Cells were separated as CD14+/CD16b− (monocytes), CD14−/CD16b+ (neutrophils) (A), CD19+ (B lymphocytes), CD3+ (T lymphocytes) (B). Slides were prepared of the sorted subpopulations. Leukocyte type was determined based on nuclear and cytoplasmic stain and morphology. (C) TNFα positive cells were identified using surface markers as above, in combination with intracellular staining for TNFα. (D) PB was stimulated with PGN (10 µg/ml) for 2, 4 and 6 hours. Cell populations were identified by surface markers and TNFα was measured by intracellular staining and flow cytometry. Two separate preparations from two donors were used; for the 6 hour point, five preparations were used. (E) PB was stimulated with PGN and the percent TNFα+monocytes (CD14+/CD16b−) was measured after 6 hours at each dose by intracellular staining and flow cytometry. Three separate preparations from two donors were used. For PGN (10 µg/ml), five preparations were used. The data for panel D and E are expressed as the mean±SEM. Statistical significance was determined by ANOVA with Bonferroni post test. *, p<0.001 *versus* 0 PGN.

To identify the cell populations within the complex cell mixture of PB that were producing TNFα, we stimulated PB with PGN and measured TNFα by intracellular cytokine staining. Unique cell surface markers were used to identify monocytes, neutrophils and lymphocytes. We first confirmed the validity of surface markers used to gate the various PB cell populations by sorting the antibody-stained PB. Sorting was based on forward scatter and side scatter properties (FSC/SSC) and surface marker-positive fluorescent gates; CD3 for T lymphocytes; CD19 for B lymphocytes; CD14 in combination with CD16b to distinguish monocytes and neutrophils. The sorted cells were stained with May-Grunwald Giemsa and identified by morphology (Figure 3A). For CD14+/CD16b− cells, 100% (243/243) showed a morphology consistent with monocytes; CD14−/CD16b+ cells, 98.5% (202/205) showed a neutrophil morphology. Likewise, CD19+ and CD3+ cells showed a lymphocyte morphology free of myeloid cells (Figure 3B). All CD3+/CD19-cells were lymphocytes (230/230) as were CD3−/CD19+ cells (258/258).

Using this gating strategy, we applied intracellular cytokine staining to determine the percentage of each unique cell population that was producing TNFα in response to *B. anthracis* PGN (Figure 3C). Surprisingly, we found that monocytes were the sole source of TNFα when PB was stimulated with PGN (10 µg/ml). Six hours after stimulation 33.2% of monocytes were positive for intracellular TNFα, compared to <1% of monocytes in unstimulated blood (Figure 3D). TNFα production was insignificant in neutrophils (CD14−/CD16b+) and T and B lymphocytes (CD3+, and CD19+) when stimulated with PGN; less than 2% of cells in these populations were positive for TNFα. PB monocytes responded in a dose-dependent manner to increasing doses of PGN from 0.1 to 10 µg/ml (Figure 3E). The percent positive at 1, 5 and 10 µg/ml of PGN was 5%, 26% and 33%, respectively. At doses above 5 µg/ml, the percent of CD14+/CD16b− monocytes positive for intracellular TNFα was significantly increased in comparison with the unstimulated samples. Thus, monocytic cells but not neutrophils or T or B lymphocytes respond to PGN derived from *B. anthracis* by producing TNFα. As an additional control, we stimulated the whole blood with PMA/ionomycin or LPS and measured cytoplasmic interferon-gamma, TNF-β or oncostatin M. We found that the T lymphocyte population responded to PMA/ionomycin with an increase in interferon-gamma while B lymphocytes produced TNF-β not shown). The neutrophil population responded to LPS with an increase in oncostatin M (not shown). Thus, these other blood cells can respond to stimuli and the failure of these other cells to respond to PGN is not for trivial reasons.

### TNFα activity is not due to endotoxin, LTA, or bacterial DNA, RNA, protein contamination of the PGN preparation

**Figure 4:**
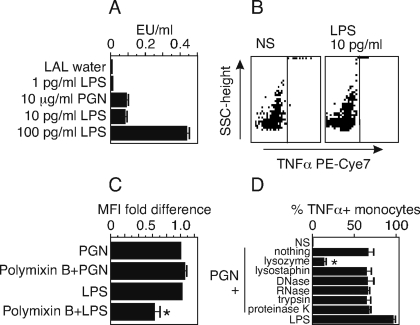
Production of TNFα is not due to endotoxin, bacterial DNA, RNA or contaminating proteins in *B. anthracis* peptidoglycan. A) The Limulus amebocyte lysate assay was used to test PGN (10 µg/ml), LPS (1 pg/ml, 10 pg/ml, 100 pg/ml) and endotoxin-free water for endotoxin activity, Endotoxin units (EU)/ml. Data are expressed as mean±SEM of 3 replicates. Statistical significance was determined for LPS (10 pg/ml) compared with PGN (10 µg/ml) by unpaired, two-tailed t test; p = 0.49, indicating no significant difference in level of endotoxin activity. (B) PB was stimulated with 10 pg/ml LPS 6 hours and TNFα positive monocytes were measured by flow cytometry. (C) PB was stimulated with PGN or LPS with and without polymixin B (PMB) (10 µg/ml) to bind endotoxin. TNFα production is expressed as fold difference±SEM of MFI of TNFα positive monocytes for 2 separate preparations on two individuals, 2 replicates each. Statistical significance was determined by ANOVA with Bonferroni post test. *, p<0.001 *versus* 1 mg/ml LPS without PMB. (D) *B. anthracis* PGN was digested with the indicted hydrolytic enzymes for 24 hours. The preparations were heat inactivated and used to stimulate PB at 10 µg/ml PGN along with an equivalent preparation of untreated PGN. The percent of TNFα positive monocytes was measured by intracellular cytokine staining. Data are expressed as the mean±SEM of three separate preparations. Statistical significance was performed on results from the enzyme treated PGN stimulations *versus* stimulation with untreated PGN by ANOVA with Bonferroni post test. *, p<0.001 *versus* untreated PGN.

The ability to stimulate TNFα production by the *B. anthracis* PGN could be due to contamination of our PGN preparation by a Gram-negative endotoxin or by DNA, RNA or proteins from *B. anthracis* that are known to stimulate TLRs in mammalian cells [15]. To test the possibility that something other than *B. anthracis* PGN was responsible for the TNFα response in PB, we examined the *B. anthracis* PGN preparation for endotoxin at the working concentration of 10 µg/ml, using the Cambrex endotoxin assay. We found that the PGN at 10 µg/ml had about 0.1 endotoxin unit (EU)/ml endotoxin activity (Figure 4A) which corresponded to 10 pg/ml of *E. coli* LPS. We then tested this amount of *E. coli* LPS to see if it would stimulate PB monocytes to produce TNFα and found that this dose failed to stimulate TNFα production (Figure 4B). To further assess a potential contribution of endotoxin, PGN stimulation of TNFα production from whole blood was performed in the presence of 10 µg/ml polymixin B. While polymixin B reduced the mean fluorescence intensity (MFI) of TNFα expression in monocytes by 44% in response to LPS, no change was detected in the response to PGN in the presence of polymixin B (Figure 4C). Thus, we conclude that the TNFα response we measured in these experiments was not due to contaminating Gram-negative endotoxin. To test for other potential TLR-stimulating material contaminating the *B. anthracis* PGN, we treated the PGN with various hydrolytic enzymes. Lysozyme digests the glycosidic bonds of the PGN backbone [23], [39]. Lysostaphin specifically digests the pentaglycine bridge of the stem peptide present in *Staphylococcus* PGN and reduces the cytokine response [39]. DNase and RNase digest CpG and bacterial RNA that could stimulate TLR9 and 3, respectively. Trypsin and proteinase K digest proteins that might contaminate our PGN preparation and could stimulate an unknown pattern recognition receptor in monocytes. Of these hydrolytic enzymes, only lysozyme was able to reduce the TNFα-stimulating ability of the PGN from *B. anthracis* (Figure 4D). None of the enzymes or buffers alone induced TNFα (data not shown). It should be noted that D-amino acids commonly present in the stem peptide are resistant to these enzymes, but D-amino acids are absent from bacterial proteins [40]. The findings indicate that the TNFα response was not due to contaminating DNA, RNA or proteins and required the intact glycan chain of PGN that is sensitive to lysozyme.

Contamination with LTA has been a factor in studies of the immunostimulatory properties of PGN preparations from Gram-positive bacteria [41], although there have been no reports of LTA in *B. anthracis* [42]. LTA is a polymeric chain composed of units of an alkane (glycerol or a monosaccharide) joined to phosphoric acid by phosphodiester bonds and hence is rich in organic phosphate [26]. We compared the phosphate content of *B. anthracis* PGN to an equivalent PGN preparation from an organism known to have LTA, *B. subtilis* [ATCC6051; [35]]. We found that the *B. anthracis* PGN had an 8-fold lower phosphate content than PGN of *B. subtilis*, 0.14 nmoles/µg of PGN vs. 1.1 nmoles/µg, respectively. We also extracted *B. anthracis* with butanol and subjected the aqueous phase to hydrophobic interaction chromatography, eluting with increasing concentrations of isopropanol. Using this procedure, we were able to detect an authentic, commercial LTA isolated from the related species *B. subtilis* using an organic phosphate assay, but no LTA was detected from *B. anthracis* extracts (data not shown). The finding suggests that *B. anthracis* lacks LTA and it is therefore unlikely that the induction of TNFα we describe above is due to LTA. Additionally, cleavage of phosphodiester bonds by HF, a procedure that efficiently hydrolyzes LTA [27], did not reduce TNFα induction by PGN (see below).

### The PGN-associated polysaccharide is not necessary for TNFα activity

**Figure 5:**
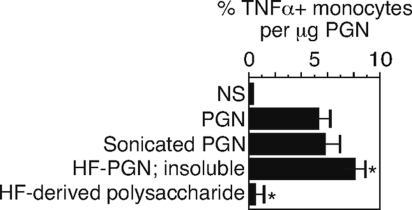
The PGN-associated polysaccharide of *B. anthracis* is not necessary for TNFα activity. *B. anthracis* PGN was sonicated and hydrolyzed with HF for 24 hours. The HF-soluble polysaccharide was separated from the insoluble peptidoglycan backbone by centrifugation. The original PGN, sonicated PGN, and HF-soluble polysaccharide and HF-insoluble PGN fractions were used to stimulate PB. The percent TNFα positive monocytes was calculated per µg of PGN (OD_600_). Data is expressed as mean±SEM of three separate preparations from 2 donors including two separate HF hydrolysis reactions of PGN. Statistical analysis was by ANOVA with Bonferroni post test of the stimulated samples *versus* PGN. *, p<0.001 *versus* PGN.

The PGN-associated polysaccharide in *B. anthracis* is unique to *B. anthracis* among several closely related species [25]. We determined whether the PGN-associated polysaccharide was necessary for TNFα induction by digesting the PGN with HF to remove the polysaccharide from the insoluble PGN. Monosaccharide analysis showed that HF digestion removed the polysaccharide from the PGN backbone (Table 1). The digestion yielded a HF-soluble fraction and the HF-insoluble fraction containing the PGN backbone. We sonicated *B. anthracis* PGN to improve its suspension during the HF digestion, and therefore also examined the effect of sonication on TNFα production by monocytes. We then tested both HF-insoluble (containing the PGN) and HF-soluble (containing the polysaccharide) fractions for their ability to induce TNFα. To compensate for a loss of mass during the HF digestion and washes, we report the data as the percent of TNFα+ monocytes per µg of solid material that remained. We found (Figure 5) that only the fraction containing the HF-digested PGN was able to induce TNFα. The loss of activity in the polysaccharide containing HF-soluble fraction was not due to sonication, since sonicated PGN retained 100% of its biological activity (Figure 5). The data indicate that the ability of *B. anthracis*-derived PGN to induce TNFα in human monocytes requires only the PGN component and not the bound polysaccharide. The fact that the HF-digested PGN was as efficacious as undigested PGN shows that LTA is not contributing to the TNFα induction, since HF also hydrolyzes any LTA that might also be present [27].

### TNF*a* is produced through a p38 MAP kinase pathway

**Figure 6:**
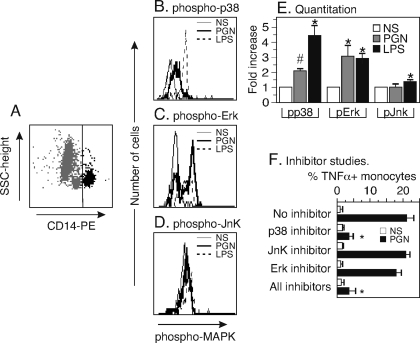
PGN stimulates the p38 MAP kinase and ERK pathways and TNFα is blocked by inhibition of p38 MAP kinase. (A) PB was stimulated with PGN (10 µg/ml) or LPS (1 µg/ml) and phospho-MAP kinases were measured by intracellular staining in CD14+ monocytes. (B) Phospho-p38 MAP kinase was measured after 15 min for the unstimulated (thin solid line), PGN (thick solid line), and LPS (dashed line). (C) Phospho p44/42 MAP kinase, 15 minutes and (D) phospho-JnK, 15 minutes. (E) Fold increase in phospho-MAP kinases for unstimulated (NS), PGN and LPS stimulation. Data are expressed as mean±SEM of three separate PB preparations. Statistical analysis was performed on MFI of stimulated PB versus MFI unstimulated PB. *, p<0.03; # p<0.005 by paired one-tailed t test. (F) PB was co-stimulated for 2 hours with PGN (10 µg/ml) with the MAP kinase inhibitors SB202190 (p38 MAP kinase inhibitor), SP600125 (JnK inhibitor) and PD 98095 (ERK inhibitor). TNFα in monocytes was measured by intracellular staining and flow cytometry. Data are expressed as mean±SEM of three separate preparations from two donors. Statistical analysis was by ANOVA with Bonferroni post test. *, p<0.01 *versus* PGN (10 µg/ml).

To explore the signal transduction pathway stimulated by PGN in monocytes and leading to TNFα production, we tested whether MAP kinases in monocytes were stimulated by PGN. As in the experiments in Figure 3, we used diluted whole PB stimulated with 10 µg/ml PGN for 15 minutes. The cells were fixed and stained with fluorochrome-labeled antibodies to CD14 to gate on monocytes, and co-stained with commercially labeled antibodies to phosphorylated forms of MAP kinase proteins: phospho-p38, phospho-p44/42 Erk kinase and phospho-Jun kinase (JnK). We gated on CD14+ cells and generated histograms of the phospho-MAP kinase levels of the unstimulated or stimulated cells. The histograms are shown in Figure 6A–D and a summary of 3 experiments is shown in Figure 6E. We found that phospho-p38 MAP kinase and phospho-Erk MAP kinases were significantly and consistently increased by 1.8 fold and 3 fold, respectively, while phospho-JnK was not increased. Thus, stimulation of monocytes with PGN causes activation of the MAP kinase pathways involving ERK and p38 but not JnK.

To explore whether activation of these MAP kinase pathway(s) are required for TNFα production, we tested the effects of the MAP kinase inhibitors SB202190 (a p38 MAP kinase inhibitor), SP600125 (a JnK inhibitor), and PD98095 (an Erk inhibitor). For these experiments, PB were stimulated with 10 µg/ml PGN in the presence of the each inhibitor. TNFα was measured by intracellular cytokine assay after 2 hours and a summary of 3 separate experiments is shown in Figure 6F. PGN stimulated about 20% of monocytes to express TNFα protein in the absence of MAP kinase inhibitors and 3.3% in the presence of 10 µM of the p38 MAP kinase inhibitor. The other MAP kinase inhibitors did not notably reduce TNFα production induced by PGN. Inhibition of Erk slightly reduced the number of TNFα positive cells but the effect was not statistically significant. We confirmed this result by repeating the PGN stimulation in the presence of inhibitors and harvesting PB supernatants for analysis by multiplex bead immunoassay as described in the Materials and Methods. Inhibition of p38 MAP kinase caused a 96% reduction in TNFα (p<0.01 by ANOVA with Bonferroni post test), while inhibition of the Erk and JnK caused no significant difference in TNFα (p>0.05, data not shown). Thus, although the ERK pathway is induced by *B. anthracis* PGN, it is not required for production of TNFα. Of the three MAP kinase pathways, the p38 pathway is critical to TNFα production.

### PGN induces expression of TNFα mRNA in PB

**Figure 7:**
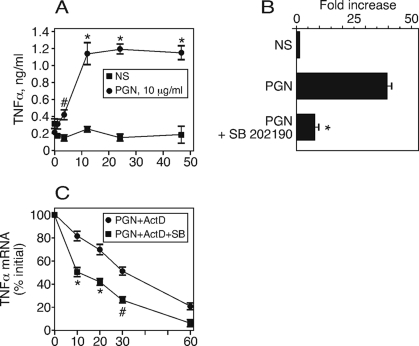
TNFα protein and mRNA is upregulated by PGN. (A) Time course of secreted TNFα induced by PGN in whole blood. PB was stimulated with PGN (10 µg/ml) and the supernatant was sampled at the indicated times. TNFα concentration was measured by ELISA. Data are expressed as mean±SEM for 3 replicates. Statistical analysis was by ANOVA with Bonferroni post test. *, p<0.001 *versus* 0 PGN; #, p<0.01 *versus* 0 PGN. (B) PB was stimulated with PGN (10 µg/ml)±SB202190 for 2 hours. RNA was isolated from leukocytes, converted to cDNA which was subjected to real-time quantitative PCR. Gene specific primers used were for TNFα and β-actin in a real-time PCR assay. Data presented are the mean±SEM of separate preparations of the TNFα/β-actin ratio from 4 individuals, 3 replicates each and normalized to the unstimulated ratio. Statistical analysis was by t test. *, p<0.01 for TNFα, PGN *versus* unstimulated; # p>0.3 for β-actin *versus* unstimulated. (C) PB was stimulated with PGN (10 µg/ml) for 3 hours. Cells were harvested to represent 100% RNA at 3 hours. To equivalent samples, actinomycin D was added immediately to block further RNA synthesis, or actinomycin+SB202190 were added to block RNA synthesis and the p38 MAP kinase pathway. Cells were harvested at 10, 20, 30, 60 minutes after these additions. RNA was isolated, cDNA was prepared and subjected to real-time quantitative PCR for TNFα as in (B). Quantity of RNA was expressed as % of RNA after 3 hours of stimulation. Data presented are the mean±SEM from 3 replicate PCR reactions from 1 individual. Statistical analysis was by ANOVA with Bonferroni post test of *, p<0.001 for PGN+ActD *versus* PGN+ActD+SB; #, p<0.01.

The appearance of TNFα in the supernatant of PGN-stimulated PB cells might be due to secretion of pre-existing protein, to increases in TNFα mRNA levels, and/or to mRNA stabilization [43]. To distinguish these possibilities, we determined whether TNFα mRNA expression is increased with PGN stimulation. We first applied a time course to determine the period after stimulation of diluted whole PB with 10 µg/ml PGN that produced the maximum TNFα protein. Maximum TNFα production occurred between 6 and 12 hours after stimulation (Figure 7A). Based on this finding, we extracted total RNA from PB six hours after stimulation and generated cDNA from total RNA. The cDNA was applied to a quantitative real-time PCR assay to measure relative levels of TNFα mRNA. We used β-actin mRNA as a standard to control for the amount of cDNA applied to the stimulated and unstimulated samples. We found a 40-fold increase in TNFα mRNA expression in the PGN-stimulated PB compared to unstimulated PB (Figure 7B). The reference gene, β-actin, was used to normalize the mRNA level since it showed no change in expression in PGN stimulation PB relative to unstimulated PB. The increase was largely blocked by inclusion of the p38 inhibitor. The result suggets that PGN stimulation induces a significant increase in TNFα mRNA in a p38-dependent way, and strongly suggest that the TNFα protein produced in response to PGN is due to nascent synthesis of TNFα. To measure the contribution of p38 to TNFα mRNA stability, we stimulated cells for 3 hours with PGN and added actinomycin D to prevent continued new mRNA synthesis. The p38 inhibitor SB 202190 [44] was added to some samples and the amount of remaining TNFα mRNA was measured by real-time quantitative PCR. We found (Figure 7C) that the TNFα mRNA decay in the presence of the p38 inhibitor was greatly accelerated relative to the decay rate in the absence of the inhibitor. These data strongly suggest that the p38 MAP kinase pathway contributes to TNFα mRNA production as well as TNFα mRNA stability to support TNFα production in PGN-stimulated monocytes.

## Discussion

We hypothesized that human PB would display a proinflammatory response upon stimulation with the *B. anthracis* PGN. We tested this hypothesis by extracting and purifying PGN, stimulating human PB and measuring secreted cytokines including TNFα, intracellular TNFα in hematopoietic cells of PB, and TNFα messenger RNA in the responding cells. Secreted TNFα was significantly increased in PB supernatant compared to unstimulated blood, with the greatest rate of accumulation of protein between 6 and 12 hours after stimulation. Surprisingly, monocytes were the major and possibly the sole source of TNFα. Intracellular TNFα and TNFα mRNA was significantly increased in monocytes when measured from 2–6 hours after stimulation. The biological activity of *B. anthracis* PGN was not due to the associated polysaccharide nor to contaminating endotoxin, or bacterial protein or nucleic acids, since it was insensitive to chemical hydrolytic or neutralizing agents that removed these TLR ligands. The production of TNFα appeared to be due to nascent mRNA and protein synthesis and required the activation of the p38 MAP kinase pathway to stabilize TNFα mRNA. The data establish that the PGN of *B. anthracis* is indeed an important endogenous toxin that is able to stimulate inflammation and the pathogenic effects associated with inflammation. When present at the high circulating amounts that are reported to occur in inhalation anthrax, the contribution of the PGN to the pathology of the infection is likely very high.

Although monocytes are the only cell type in PB that produce an early TNFα response to *B. anthracis* PGN, neutrophils and lymphocytes are capable of producing TNFα in response to various stimulants. T lymphocytes produce TNFα after infection with influenza virus [45], and B lymphocytes produce TNFα after stimulation of the B cell antigen receptor and CD40 [46]. Human neutrophils produced TNFα in response to LTA from *S. aureus* [47] and *E. coli* LPS [48]. The fact that our flow cytometry data showed that neutrophils are not a significant source of TNFα induced by our PGN preparation is additional evidence that the activity in the *B. anthracis* PGN preparation described here is not due to LTA contamination. Thus, although other hematopoietic cells in PB are capable of producing TNFα in response to various infectious agents, only monocytes respond to *B. anthracis* PGN. This fact may be due to the expression of a unique subset of Toll-like or other pattern recognition receptors on monocytes that are absent from lymphocytes or neutrophils. The unique ability of monocytes to mount an early inflammatory response to *B. anthracis* PGN has important implications for therapies that aim to block *B. anthracis* sepsis at the stage when the vegetative cell has disseminated to the PB. Monocytes are the most important target for reducing the inflammatory response that may lead to septic shock.

An earlier report showed isolated monocytes pre-activated with interferon-γ produce TNFα in response to non-purified cell wall extracts of *B. anthracis* [28]. However, our study offers several improvements. First, *ex vivo* stimulation of whole unmanipulated peripheral blood, relative to stimulation of isolated and pre-activated monocytes, provides a more accurate representation of the *in vivo* responses. Indeed, experiments have shown that the presence of autologous red blood cells with cultured blood-derived lymphocytes and monocytes altered the production of cytokines relative to that produced by isolated lymphocytes and monocytes [49]. TNFα and interferon-γ were reported to be produced at higher levels when the monocytes are present in whole peripheral blood environment relative to isolated monocytes [50]. Second, supplements added to media used for culturing isolated cells could skew the biological response of the cells. For example, stimulation of T cells in serum-supplemented medium enhanced production of IL-2 and IL-4 and while depressing secretion of IL-4, IL-5 and interferon-γ, compared with cells in serum-free media [51]. In our experiments, the cells are resting and are present in the endogenous milieu of whole blood. This situation more closely mimics the state of the monocytes *in vivo* when they encounter the cell wall of *B. anthracis* as the organism enters the peripheral blood during an active anthrax infection. Lastly, our PGN preparation was extensively purified and tested for numerous contaminants than the preparation previously used to stimulate monocytes [28]. Specifically, we demonstrate using a test for endotoxin contamination, the use of polymixin B, and the application of a variety of hydrolytic enzymes and chemicals, that the biologically active material in our purified PGN preparation was PGN and not other bacterial substance such as bacterial DNA, RNA, or protein, or LTA.

Our data indicates that the glycan chain of the PGN is an important factor in the induction of TNFα in human monocytes. Hydrolysis of the glycosidic linkages of the PGN murein core with lysozyme caused a significant reduction in the TNFα production stimulated by PGN. Digestion with lysostaphin, which hydrolyzes the interpeptide bridge connecting the stem peptides in *S. aureus* PGN, did not reduce TNFα. This finding suggests that *B. anthracis* PGN lacks a pentaglycine bridge linking the stem peptide that is present in *S. aureus* PGN. Other Gram-positive organisms use instead a direct crosslink between diaminopimelic acid and another amino acid in the stem peptide of the adjacent glycan chain [21], [23]. We isolated diaminopimelic acid in the *B. anthracis* PGN preparation, consistent with lack of a pentaglycine bridge. The presence of significant aspartic acid levels (see text) may suggest a bridge containing aspartic acid rather than a direct cross-link with diaminopimelic acid. Other protease digestions of PGN with trypsin and proteinase K likewise did not reduce TNFα production. The amino acid sequence of the stem peptide in *B. anthracis* PGN is not known, thus its protease sensitivity is unclear. However, other proteins covalently associated with PGN should be susceptible to digestion by trypsin or proteinase K. The fact that protease digestion did not affect the biological activity of *B. anthracis* PGN is consistent with our interpretation that the PGN glycan chain and/or stem peptide itself is the biological agent that stimulates proinflammatory cytokine production, rather than potential bacterial proteins associated with it.

The PGN of *B. anthracis* is known to have a major polysaccharide bound to it by a phosphodiester bond that can be released by treatment with HF [25], [34], [52]. The polysaccharide consists of galactose, GLcNac and MurNac in a 3∶2∶1 ratio [25], [34] or 10∶2∶1 ratio [52]. Treatment of *B. anthracis* PGN with HF isolated a polysaccharide containing glucosamine, galactose and mannosamine in a ratio of 5∶3∶1 (Table 1). Hydrolysis of the polysaccharide with HCL or trifluoroacetic acid removes the acetyl groups from these monosaccharides, so N-acetyl glucosamine appears as glucosamine and N-acetyl mannosamine as mannosamine in our analysis. Our results are consistent with previous studies [25], [34], [52] as far as the major monosaccharides present in the polysaccharide. Our result differs in that glucosamine is present in greater quantity relative to galactose. This difference may have resulted from a difference in hydrolysis methods or analytical methods. Alternatively, variations in growth conditions for *B. anthracis* may alter the composition of the major polysaccharide in vegetative cells. In any case, although the authors of an earlier study of *B. anthracis* polysaccharide speculated that it may be proinflammatory [25], the polysaccharide we isolated had no detectable biological activity. Our results are consistent with previous evidence from *S. aureus* that the proinflammatory properties peptidoglycans depend on the intact glycan backbone of PGN [39], [53].

Pathogen components including *B. anthracis* PGN and Gram-negative LPS are sensed by the innate immune system through TLRs. There is evidence that TLR2 and TLR6 recognize PGN of Gram-positive organisms [15]. However, other studies have shown that the ability of TLR2 to engage Gram-positive PGN is reduced by trypsin digestion, suggesting that the TLR2 ligand might be a contaminating bacterial protein or a trypsin-sensitive stem peptide [54]. NOD2 has been shown to recognize lysozyme-digested PGN of *S. aureus* but not polymeric PGN [54]. Some studies report that N-acetylmuramic acid carrying two stem peptides, MurNac-L-Ala-D-isoGln, is recognized by NOD2 [18] and induces proinflammatory cytokines, but the response depends on the bacterial species from which the ligand is isolated [55]. We are currently trying to identify the PGN receptor on human monocytes. However, we note our finding (Figure 4) that human monocytes recognize *B. anthracis* PGN digested with trypsin and proteinase K, but not lysozyme-digested PGN. These data suggest the unidentified human *B. anthracis* PGN receptor recognizes polymeric PGN to induce TNFα, but does not exclude a second receptor that can recognize digested products of PGN.

A poly-D glutamic acid capsule provides *B. anthracis* with resistance to phagocytosis by host myeloid cells [9]. It could be argued that the poly-D-glutamic acid capsule prevents the innate immune system from contacting the PGN. However, there are two studies that indicate the vegetative cell wall of *B. anthracis* is indeed exposed to the immune system, despite the presence of the capsule. First, sera from animals vaccinated with *B. anthracis* Sterne strain were able to stain the ends and septa of encapsulated Ames strain *B. anthracis* obtained from bacteremic pig blood [56]. Since antibodies in sera are accessible to encapsulated *B. anthracis*, the PGN in the encapsulated *B. anthracis* may similarly be exposed to monocyte pattern recognition receptors as it circulates. Second, the PGN is processed as an antigen in animals during an active infection with encapsulated *B. anthracis*. Rabbits immunized with *B. anthracis* Vollum strain, having the capsule and toxin, form antibody titers to a variety of cell wall proteins, covalently and non-covalently bound, in equal levels with antibodies to LF and secreted proteins [57]. This finding indicates that the capsule does not prevent components of the cell wall from exposure to TLRs, NOD receptors, or other receptors on cells of the immune system.

Inhibition of p38 MAP kinase provided a complete block of TNFα production from PGN-stimulated monocytes. Studies on LPS-induced TNFα production suggested the p38 pathway affected translation but not transcription of TNFα mRNA since p38 inhibition blocked LPS-triggered TNFα protein but not mRNA production [58]. The notion that p38 regulates TNFα translation but not transcription is consistent with other reports showing that TNFα mRNA was stabilized by activation of p38 MAP kinase [59]. In addition to PGN, the secreted LT is another factor affecting p38 MAP kinase during anthrax infection. LT inactivates p38 MAP kinase by cleaving the upstream MAP kinase kinases [60] and LT cleaves MAP/ERK kinase-1 [61]. The production of LT may be an evolutionary strategy by *B. anthracis* to prevent production of proinflammatory cytokine by the host immune cells. Further dissection of the regulatory activity of individual virulence factors on proinflammatory cytokines will likely illuminate mechanism of sepsis, septic shock and host death in *B. anthracis*.

Traditional notions about anthrax disease have focused on LT as the cause of toxic shock and death. However, toxin-null *B. anthracis* stains are lethal in mice [11]. If toxic shock is not a major cause of death, inflammation and septic shock may contribute. A significant inflammatory response, including secreted TNFα, is observed in baboons challenged with anthrax, although the contribution of toxin to this response has not been determined [62]. TNFα is a major contributor to sepsis, especially if not modulated by anti-inflammatory mediators [63]. The secretion of TNFα from monocytes stimulated with PGN is consistent with a model of anthrax as a septic disease. Our results define p38 MAP kinase and monocytes as a potential therapeutic target for intervening in the *B. anthracis* inflammatory process that leads to septic shock. Several p38 MAP kinase inhibitors are being explored as agents against arthritis and septic shock [59], [64]. These inhibitors may be useful adjuvant therapies in the treatment of anthrax.

## References

[1] Cote CK, Van Rooijen N, Welkos SL (2006). Roles of macrophages and neutrophils in the early host response to Bacillus anthracis spores in a mouse model of infection.. Infect Immun.

[2] Rhie GE, Roehrl MH, Mourez M, Collier RJ, Mekalanos JJ (2003). A dually active anthrax vaccine that confers protection against both bacilli and toxins.. Proc Natl Acad Sci U S A.

[3] Dixon TC, Meselson M, Guillemin J, Hanna PC (1999). Anthrax.. N Engl J Med.

[4] Guarner J, Jernigan JA, Shieh WJ, Tatti K, Flannagan LM (2003). Pathology and pathogenesis of bioterrorism-related inhalational anthrax.. Am J Pathol.

[5] Jernigan JA, Stephens DS, Ashford DA, Omenaca C, Topiel MS (2001). Bioterrorism-related inhalational anthrax: the first 10 cases reported in the United States.. Emerg Infect Dis.

[6] Grinberg LM, Abramova FA, Yampolskaya OV, Walker DH, Smith JH (2001). Quantitative pathology of inhalational anthrax I: quantitative microscopic findings.. Mod Pathol.

[7] Standiford TJ (2000). Anti-inflammatory cytokines and cytokine antagonists.. Curr Pharm Des.

[8] Hessle CC, Andersson B, Wold AE (2005). Gram-positive and Gram-negative bacteria elicit different patterns of pro-inflammatory cytokines in human monocytes.. Cytokine.

[9] Scorpio A, Chabot DJ, Day WA, O'Brien DK, Vietri NJ (2007). Poly-gamma-glutamate capsule-degrading enzyme treatment enhances phagocytosis and killing of encapsulated Bacillus anthracis.. Antimicrob Agents Chemother.

[10] Koehler TM (2002). Bacillus anthracis genetics and virulence gene regulation.. Curr Top Microbiol Immunol.

[11] Heninger S, Drysdale M, Lovchik J, Hutt J, Lipscomb MF (2006). Toxin-deficient mutants of Bacillus anthracis are lethal in a murine model for pulmonary anthrax.. Infect Immun.

[12] Cleret A, Quesnel-Hellmann A, Mathieu J, Vidal D, Tournier JN (2006). Resident CD11c+ lung cells are impaired by anthrax toxins after spore infection.. J Infect Dis.

[13] Coffey RG (1992). Effects of cyclic nucleotides on granulocytes.. Immunol Ser.

[14] Moayeri M, Haines D, Young HA, Leppla SH (2003). Bacillus anthracis lethal toxin induces TNF-alpha-independent hypoxia-mediated toxicity in mice.. J Clin Invest.

[15] Nakao Y, Funami K, Kikkawa S, Taniguchi M, Nishiguchi M (2005). Surface-expressed TLR6 participates in the recognition of diacylated lipopeptide and peptidoglycan in human cells.. J Immunol.

[16] Liu C, Xu Z, Gupta D, Dziarski R (2001). Peptidoglycan recognition proteins: a novel family of four human innate immunity pattern recognition molecules.. J Biol Chem.

[17] Girardin SE, Boneca IG, Carneiro LA, Antignac A, Jehanno M (2003). Nod1 detects a unique muropeptide from gram-negative bacterial peptidoglycan.. Science.

[18] Girardin SE, Boneca IG, Viala J, Chamaillard M, Labigne A (2003). Nod2 is a general sensor of peptidoglycan through muramyl dipeptide (MDP) detection.. J Biol Chem.

[19] Chamaillard M, Hashimoto M, Horie Y, Masumoto J, Qiu S (2003). An essential role for NOD1 in host recognition of bacterial peptidoglycan containing diaminopimelic acid.. Nat Immunol.

[20] Girardin SE, Travassos LH, Herve M, Blanot D, Boneca IG (2003). Peptidoglycan molecular requirements allowing detection by Nod1 and Nod2.. J Biol Chem.

[21] Schleifer KH, Kandler O (1972). Peptidoglycan types of bacterial cell walls and their taxonomic implications.. Bacteriol Rev.

[22] Vollmer W, Blanot D, de Pedro MA (2008). Peptidoglycan structure and architecture.. FEMS Microbiol Rev.

[23] Fournier B, Philpott DJ (2005). Recognition of Staphylococcus aureus by the innate immune system.. Clin Microbiol Rev.

[24] Zhang X, Rimpilainen M, Simelyte E, Toivanen P (2001). Characterisation of Eubacterium cell wall: peptidoglycan structure determines arthritogenicity.. Ann Rheum Dis.

[25] Choudhury B, Leoff C, Saile E, Wilkins P, Quinn CP (2006). The structure of the major cell wall polysaccharide of Bacillus anthracis is species-specific.. J Biol Chem.

[26] Iwasaki H, Shimada A, Ito E (1986). Comparative studies of lipoteichoic acids from several Bacillus strains.. J Bacteriol.

[27] Morath S, Geyer A, Hartung T (2001). Structure-function relationship of cytokine induction by lipoteichoic acid from Staphylococcus aureus.. J Exp Med.

[28] Popov SG, Villasmil R, Bernardi J, Grene E, Cardwell J (2002). Effect of Bacillus anthracis lethal toxin on human peripheral blood mononuclear cells.. FEBS Lett.

[29] Rosenthal RS, Dziarski R (1994). Isolation of peptidoglycan and soluble peptidoglycan fragments.. Methods Enzymol.

[30] Clarke AJ (1993). Compositional analysis of peptidoglycan by high-performance anion-exchange chromatography.. Anal Biochem.

[31] Hardy MR, Townsend RR (1994). High-pH anion-exchange chromatography of glycoprotein-derived carbohydrates.. Methods Enzymol.

[32] Chakrabarty K, Wu W, Booth JL, Duggan ES, Coggeshall KM (2006). Bacillus anthracis spores stimulate cytokine and chemokine innate immune responses in human alveolar macrophages through multiple mitogen-activated protein kinase pathways.. Infect Immun.

[33] Chow S, Hedley D, Grom P, Magari R, Jacobberger JW (2005). Whole blood fixation and permeabilization protocol with red blood cell lysis for flow cytometry of intracellular phosphorylated epitopes in leukocyte subpopulations.. Cytometry A.

[34] Ekwunife FS, Singh J, Taylor KG, Doyle RJ (1991). Isolation and purification of cell wall polysaccharide of Bacillus anthracis (delta Sterne).. FEMS Microbiol Lett.

[35] Morath S, Geyer A, Spreitzer I, Hermann C, Hartung T (2002). Structural decomposition and heterogeneity of commercial lipoteichoic Acid preparations.. Infect Immun.

[36] Gao JJ, Xue Q, Zuvanich EG, Haghi KR, Morrison DC (2001). Commercial preparations of lipoteichoic acid contain endotoxin that contributes to activation of mouse macrophages in vitro.. Infect Immun.

[37] Livak KJ, Schmittgen TD (2001). Analysis of relative gene expression data using real-time quantitative PCR and the 2(-Delta Delta C(T)) Method.. Methods.

[38] Garrido G, Delgado R, Lemus Y, Rodriguez J, Garcia D (2004). Protection against septic shock and suppression of tumor necrosis factor alpha and nitric oxide production on macrophages and microglia by a standard aqueous extract of Mangifera indica L. (VIMANG). Role of mangiferin isolated from the extract.. Pharmacol Res.

[39] Myhre AE, Stuestol JF, Dahle MK, Overland G, Thiemermann C (2004). Organ injury and cytokine release caused by peptidoglycan are dependent on the structural integrity of the glycan chain.. Infect Immun.

[40] Tugyi R, Uray K, Ivan D, Fellinger E, Perkins A (2005). Partial D-amino acid substitution: Improved enzymatic stability and preserved Ab recognition of a MUC2 epitope peptide.. Proc Natl Acad Sci U S A.

[41] Girardin SE, Philpott DJ (2004). Mini-review: the role of peptidoglycan recognition in innate immunity.. Eur J Immunol.

[42] Fouet A, Mesnage S (2002). Bacillus anthracis cell envelope components.. Curr Top Microbiol Immunol.

[43] Mahtani KR, Brook M, Dean JL, Sully G, Saklatvala J (2001). Mitogen-activated protein kinase p38 controls the expression and posttranslational modification of tristetraprolin, a regulator of tumor necrosis factor alpha mRNA stability.. Mol Cell Biol.

[44] Underwood DC, Osborn RR, Kotzer CJ, Adams JL, Lee JC (2000). SB 239063, a potent p38 MAP kinase inhibitor, reduces inflammatory cytokine production, airways eosinophil infiltration, and persistence.. J Pharmacol Exp Ther.

[45] Crowe SR, Miller SC, Shenyo RM, Woodland DL (2005). Vaccination with an acidic polymerase epitope of influenza virus elicits a potent antiviral T cell response but delayed clearance of an influenza virus challenge.. J Immunol.

[46] Duddy ME, Alter A, Bar-Or A (2004). Distinct profiles of human B cell effector cytokines: a role in immune regulation?. J Immunol.

[47] Hattar K, Grandel U, Moeller A, Fink L, Iglhaut J (2006). Lipoteichoic acid (LTA) from Staphylococcus aureus stimulates human neutrophil cytokine release by a CD14-dependent, Toll-like-receptor-independent mechanism: Autocrine role of tumor necrosis factor-[alpha] in mediating LTA-induced interleukin-8 generation.. Crit Care Med.

[48] Dubravec DB, Spriggs DR, Mannick JA, Rodrick ML (1990). Circulating human peripheral blood granulocytes synthesize and secrete tumor necrosis factor alpha.. Proc Natl Acad Sci U S A.

[49] Kalechman Y, Herman S, Gafter U, Sredni B (1993). Enhancing effects of autologous erythrocytes on human or mouse cytokine secretion and IL-2R expression.. Cell Immunol.

[50] De Groote D, Zangerle PF, Gevaert Y, Fassotte MF, Beguin Y (1992). Direct stimulation of cytokines (IL-1 beta, TNF-alpha, IL-6, IL-2, IFN-gamma and GM-CSF) in whole blood. I. Comparison with isolated PBMC stimulation.. Cytokine.

[51] Daynes RA, Dowell T, Araneo BA (1991). Platelet-derived growth factor is a potent biologic response modifier of T cells.. J Exp Med.

[52] Mesnage S, Fontaine T, Mignot T, Delepierre M, Mock M (2000). Bacterial SLH domain proteins are non-covalently anchored to the cell surface via a conserved mechanism involving wall polysaccharide pyruvylation.. Embo J.

[53] Kengatharan KM, De Kimpe S, Robson C, Foster SJ, Thiemermann C (1998). Mechanism of gram-positive shock: identification of peptidoglycan and lipoteichoic acid moieties essential in the induction of nitric oxide synthase, shock, and multiple organ failure.. J Exp Med.

[54] Travassos LH, Girardin SE, Philpott DJ, Blanot D, Nahori MA (2004). Toll-like receptor 2-dependent bacterial sensing does not occur via peptidoglycan recognition.. EMBO Rep.

[55] Nagao S, Nakanishi M, Kutsukake H, Yagawa K, Kusumoto S (1990). Macrophages are stimulated by muramyl dipeptide to induce polymorphonuclear leukocyte accumulation in the peritoneal cavities of guinea pigs.. Infect Immun.

[56] Ezzell JW, Abshire TG (1988). Immunological analysis of cell-associated antigens of Bacillus anthracis.. Infect Immun.

[57] Gat O, Grosfeld H, Ariel N, Inbar I, Zaide G (2006). Search for Bacillus anthracis potential vaccine candidates by a functional genomic-serologic screen.. Infect Immun.

[58] Young P, McDonnell P, Dunnington D, Hand A, Laydon J (1993). Pyridinyl imidazoles inhibit IL-1 and TNF production at the protein level.. Agents Actions.

[59] Campbell J, Ciesielski CJ, Hunt AE, Horwood NJ, Beech JT (2004). A novel mechanism for TNF-alpha regulation by p38 MAPK: involvement of NF-kappa B with implications for therapy in rheumatoid arthritis.. J Immunol.

[60] Park JM, Greten FR, Li ZW, Karin M (2002). Macrophage apoptosis by anthrax lethal factor through p38 MAP kinase inhibition.. Science.

[61] Ribot WJ, Panchal RG, Brittingham KC, Ruthel G, Kenny TA (2006). Anthrax lethal toxin impairs innate immune functions of alveolar macrophages and facilitates Bacillus anthracis survival.. Infect Immun.

[62] Stearns-Kurosawa DJ, Lupu F, Taylor FB, Kinasewitz G, Kurosawa S (2006). Sepsis and pathophysiology of anthrax in a nonhuman primate model.. Am J Pathol.

[63] Walley KR, Lukacs NW, Standiford TJ, Strieter RM, Kunkel SL (1996). Balance of inflammatory cytokines related to severity and mortality of murine sepsis.. Infect Immun.

[64] Kaminska B (2005). MAPK signalling pathways as molecular targets for anti-inflammatory therapy–from molecular mechanisms to therapeutic benefits.. Biochim Biophys Acta.

